# Evaluation of immunomodulatory effects of *Boswellia sacra* essential oil on T-cells and dendritic cells

**DOI:** 10.1186/s12906-020-03146-5

**Published:** 2020-11-19

**Authors:** Alia M. Aldahlawi, Amani T. Alzahrani, Mohamed F. Elshal

**Affiliations:** 1 Department of Biological Sciences, Faculty of Sciences, King Abdulaziz University, Jeddah, Saudi Arabia; 2 Immunology Unit, King Fahad Medical Research Center, King Abdulaziz University, Jeddah, Saudi Arabia; 3 Molecular Biology Department, Genetic Engineering and Biotechnology Institute, University of Sadat City, Sadat City, Egypt

**Keywords:** Dendritic cells, *Boswellia sacra*, Essential oil, Differentiation, Maturation, And tolerance

## Abstract

**Background:**

*Boswellia sacra* resin has been commonly used as analgesic, antimicrobial, and anti-inflammatory properties, which reflect its immunomodulatory activity. Dendritic cells (DCs) are specialized antigen-presenting cells (APCs) and sentinel cells that regulate the immune response. This study aims at investigating whether crude essential oil extracted from *Boswellia sacra* resin (BSEO), has a potential effect on the phenotype and functions of human monocyte-derived DCs.

**Methods:**

Oil extract from the resin of *Boswellia sacra* was prepared by hydrodistillation using a custom made hydrodistiller. BSEO-mediated cell viability has been initially studied on human skin dermis cells (HSD) and DC precursors using quantitative and qualitative assays before applying on DCs. Human DCs were generated from differentiated peripheral blood monocytes cultured in media containing both GM-CSF and IL-4. DCs were exposed to 5 μg/mL or 10 *μ*g/mL of BSEO in vitro. Morphological, phonotypical, and functional properties studied with microscopy, flow cytometry, and ELISA.

**Results:**

Crude BSEO was found to interfere with the maturation and differentiation of DCs from precursor cells in the presence or absence of lipopolysaccharide (LPS). BSEO-treated DCs, cultured in the presence of LPS, reduced the ability of allogeneic T cells to proliferate compared to that co-cultured with LPS-stimulated DCs only. In addition, the endocytic capacity and secretion of IL-10 by DCs treated with BSEO was enhanced in comparison to LPS treated cells. Analysis of the chemical composition of BESO using GC-MS (Clarus 500 GC/MS, PerkinElmer, Shelton, CT) revealed the presence of compounds with several biological activities including antibacterial, antioxidant, and anti-inflammatory properties.

**Conclusion:**

Results indicated that BSEO deviates the differentiation of monocytes into immature DCs. Furthermore, stimulation of immature DCs with BSEO was unable to generate full DC maturation. However, these findings may potentially be employed to generate DCs with tolerogenic properties that are able to induce tolerance in diseases with hypersensitivity, autoimmunity as well as transplantation.

## Background

Burseraceae is one of the most popular aromatic plants used in incense, perfume, and as a preservative. The genus Boswellia belongs to the Burseraceae botanical family, well-known by the oilresin exuded from incisions in the trunk of the tree. It has a prolonged history of use in traditional medicines to mitigate or cure inflammatory diseases such as rheumatoid arthritis [1]. Moreover, *Boswellia sp*. includes *Boswellia sacra, Boswellia carteri,* and *Boswellia serrata*, which demonstrate substantial applications in research. The pharmacological properties of *Boswellia sp*. have also been widely investigated both in vitro and in vivo (animals or humans) [2]. The resin’s anti-inflammatory activity may be attributed to its potential to regulate multiple inflammatory mediators including cytokines, and target various signal transductions [3]. Furthermore, *Boswellia sp*. essential oil is a volatile constituent, which is mostly used in aromatherapy. Research has tried to identify and elucidate the pharmacological properties of *Boswellia sp*. essential oil [4].

Dendritic cells (DCs) are one of the three mononuclear phagocyte system known for their exclusive characteristics [5]. DCs contain distinct subpopulations critically essential for the generation of multiple functions in the immune system, which determine the outcome of immune responses. Immature DCs (imDCs) are located in the peripheral tissues where they work as sentinels and facilitate immune tolerance [6]. After DCs are activated, they become mature DCs (mDCs), which critically required for priming naïve T lymphocytes. Upon activation, naïve T cells start to proliferate and differentiate into effector cells, which are needed for the promotion of the adaptive immune response against pathogens. On the other hand, the production of immune tolerance is crucial for the maintenance of self-tolerance under steady state conditions [7, 8].

Modulation of DCs with plant-derived natural products was documented in several scientific articles. Generally, the plant and their constituents may enhance the differentiation of DCs from precursors [9], or modulate DC functions to either induce or suppress immune responses [10, 11]. To achieve such modulation, DC undergoes several phenotypical and functional changes. For instance, changes lead to up or downregulation of DC maturation markers including CD80, CD86, CD83, CD40, and HLA-DR, phagocytic activity, cytokine production, and the ability to activate CD4^+^ T cells. For Example, *Vigna angularis* (an extract from Azuki bean), pinecone extract, and *Mucuna pruviens* var. exhibited differentiation and maturation of DCs in vitro [9, 12, 13]. Studies demonstrated that changes in the functional status of DCs may bind to pattern recognition receptors, therefore could be useful targets for infectious disease therapy. Accordingly, it has been reported that both soybean and peanut agglutinin were agonists for TLR4 in humans [14].

Bearing in mind the powerful role of DCs functions in the immune system, we investigated the efficacy of using crude *Boswellia sacra* essential oil (BSEO) in the induction of DCs modulation. Therefore, the aim of the present study is to explore the impact of BSEO on human monocyte-derived dendritic cell differentiation, maturation, and functional activities.

## Methods

### Media and reagents

Cells were grown in RPMI-1640 or DMEM complete growth media containing Heat-inactivated fetal bovine serum (FBS) (Gibco, USA), and Penicillin-streptomycin solution (Pen/Strep) (HyClone, South Logan, USA). Both Phosphate-buffered saline (PBS) and Hanks’ balanced salt solution (HBSS) were obtained from UFC Biotech (KSA). Lymphoprep™ - 1.077 g/mL was purchased from Axis-Shield PoC AS (Norway). Purified *Escherichia coli* LPS and Dimethyl Sulfoxide (DMSO)-1.10 g/mL (Sigma-Aldrich®, St. Louis, USA) was used. Vitamin D3 was purchased from Nature Made (USA). All CCR7, CD83, CD80, CD14, CD71 recombinant monoclonal antibodies, recombinant human interleukin 4 (IL-4), and granulocyte-macrophage colony-stimulating factor (GM-CSF) were obtained from BioLegend® (San Diego, California). CD3 was purchased from Invitrogen (Carlsbad, California). Isotype control, CD11c, and CD86 recombinant monoclonal antibodies were purchased from R&D systems (Minneapolis, MN, USA). Lithium Heparin tubes were from Xinle sci&tech co., ltd. (China). Magnesium Sulphate anhydrous (anh. MgSO4) (M.W. =120, 37) was purchased from Panreac Quimica SA, Barcelona, Spain. Camptothecin (CPT) (Sigma Aldrich®, St. Louis, USA) was used.

### Preparation of *Boswellia sacra* essential oil (BSEO)

*Boswellia sacra* oleogum resin was purchased from Muttrah Souq in Muscat city, the capital of the Sultanate of Oman. Crude BSEO was extracted via hydrodistillation done using a regular hydrodistiller. The oleogum resins (100 g) were mixed with 500 mL distilled water and heated at 55 °C until thick solution was formed [15]. Then, the temperature of the hydrodistiller was increased up to 78 °C and remained for 3 h. The resulting mixture was filtered using a 0.22 μm filter (CHMLAB Group 08205, Barcelona (SPAIN), EEC). Finally, the crude essential oil layer was separated manually using a sterilized plastic dropper. The collected essential oil was dried over anhydrous MgSO4. The harvested essential oil was stored in sealed vials at − 80 °C until use. The stock solution of BSEO was prepared by dissolving in DMSO (1:1) to obtain an initial concentration of 25 mg/mL. Then, the stock solution was diluted in culture media to get the concentrations at 5 μg/mL, 10 *μ*g/mL, 15 *μ*g/mL, 20 *μ*g/mL, and 50 *μ*g/mL.

### Cell line culture

Normal human skin dermis cell line (HSD) was obtained from Alzamil chair for cancer research and ethically approved by King Fahad Medical Center, Jeddah, KSA. HSD cell line was cultured in complete DMEM growth medium at a density of 1 × 10^5^ cells/well and 1.5 mL/well in 12-well plates. Cells were grown in a 5% CO_2_ incubator at 37 °C in a humidified atmosphere. It was passaged twice weekly or when cells were about 70–80% confluence [16].

### DCs differentiation and maturation

The present study was approved by the research ethics committee and performed at the Immunology Unit at KFMRC, KAU. DCs isolation and differentiation were performed as reported by others [17]. Briefly, Heparinized human peripheral blood was diluted in HBSS, and Peripheral blood mononuclear cells (PBMCs) were separated by Lymphoprep density gradient centrifugation. Cells were washed and resuspended in red blood cell lysis buffer then washed twice with HBSS. A total of 50 × 10^6^ PBMCs were resuspended in RPMI-1640 medium and plated in a 12 well plate at a density of 5 × 10^6^ cells/mL and allowed to adhere for 2 h at 37 °C in a 5% CO_2_ incubator. Non-adherent cells were gently removed by washing several times with warm RPMI-1640 medium and the remaining adherent cells (monocytes) were used or cultured for further investigations. For DCs differentiation, adherent cells were cultivated in complete RPMI-1640 media containing IL-4 (50 ng/mL) and GM-CSF (100 ng/mL) for 7 days. Cultured cells were further fed on day three with complete RPMI-1640 media supplemented with the same concentration of cytokines.

### Cell viability assay

HSD cells (at density of 1 × 10^5^ cells/well and 1.5 mL/well in 12-well plates) and adherent monocytes were treated with crude BSEO at concentrations of 5 μg/mL, 10 *μ*g/mL, 15 *μ*g/mL, 20 *μ*g/mL, and 50 *μ*g/mL. DMSO (< 0.2% that had no toxic effect on cells) was used as control. After 24 h, cells were collected, washed, and resuspended in PBS. Cytotoxicity was determined by the trypan blue exclusion test by mixing an equal volume of 0.4% trypan blue dye with the cell suspension [18]. Cellular viability was determined using an inverted microscope (Nikon eclipse Ti, Tokyo, Japan) supplemented with digital camera software (NIS-Elements F 3.2, Nikon, Tokyo, Japan.

### DCs differentiation and maturation assays

To investigate the capability of BSEO to induce differentiation of monocytes into imDCs, 5 μg/mL or 10 *μ*g/mL BSEO were added separately to adherent monocytes in the absence of cytokines. In parallel, adherent monocytes were also cultured in the presence of cytokines for comparison purposes. For the determination of DC maturation, cells were stimulated for further 24 h with either BSEO at 5 μg/mL or 10 *μ*g/mL. Vitamin D3 at 12.5 ng/mL was used to induce tolerogenic DCs. Mature DCs were achieved by adding LPS (1 μg/mL) as a positive control and imDCs were left unstimulated or stimulated with 1% of DMSO. The effects of BSEO were also investigated on 1 *μ*g/mL LPS stimulated DCs [19].

### Flow cytometry

All BSEO treated and untreated cells were analyzed by Navios flow cytometer (Beckman Coulter Life Science, USA) following induction of differentiation or maturation. Cells were harvested, washed, and counted before staining. Then cells were stained with recombinant monoclonal antibodies including APC-conjugated anti-CD14, FITC-conjugated anti-CD11c, anti-HLA-DR, anti-CD83, anti-CD80, anti-CD86, and anti-CCR7 for 30 min at 4 °C. For negative control staining, appropriate conjugated isotypes were used. Following staining, all cells were washed with cold PBS and resuspended in 500 μL of PBS. Data were acquired and analyzed by Navios software (Beckman coulter life science, USA) [19].

### Apoptosis assay

To determine the effects of BSEO treatment on the induction of apoptosis or necrosis in DCs, apoptosis detection kit (TACS®Annexin V-FITC, Trevigen, Gaithersburg, USA) was used and performed according to the manufacturer’s guidelines. CPT was used as an apoptosis inducer at a concentration of 10 mM for 4 h and purchased from Sigma Aldrich®,St. Louis, USA. Briefly, cells were collected, washed once, and resuspended in 100 μL of Annexin-V reagent. Cells were left in the dark for 15 min at room temperature. Finally, 400 μL of 1X binding buffer was added to stained cells and acquired by Navios flow cytometry (Beckman coulter life science, USA) within 1 h and analyzed using Navios software [20]. Ten *μ*L of PI was added to the cells prior to analysis by flow cytometry. Consequently, viable cells do not take any color (Annexin V−/PI-), early apoptotic cells are green (Annexin V+/PI-), late apoptotic cells are green and orange (Annexin V+/PI+), and necrotic cells are orange (Annexin V−/PI+).

### Mixed lymphocytes reaction (MLR) assay

For the induction of allogenic MLR, stimulated DCs (as a stimulator) were harvested, washed, and co-cultured with fresh allogeneic PBMCs (as a responder) in a ratio 1:10 for 3 days at 37 °C in a 5% CO_2_ incubator. Allogeneic PBMCs were used as a source of T cells. For comparison, imDCs and PBMCs were cultured separately and used as control. After 3 days, cells were washed, counted, and stained with anti-CD3 plus anti-CD71 for 30 min at 4 °C. Later, cells were washed twice and re-suspended in 200 μL PBS supplemented with 2% FBS. Finally, stained cells were acquired and analyzed by Navios flow cytometry and software (Beckman coulter life science, USA) [21].

### Endocytosis assay

To determine the internalization capability of stimulated and unstimulated DCs, cells were collected and counted prior to staining. Harvested cells were washed and incubated with prewarm RPMI-1640 media without serum containing 1 mg/mL FITC-dextran (MW 40000 Da powder, UFC Biotech, KSA) at either 4 °C (negative control) or 37 °C (positive control) for 3 h. Then, cells were washed with 5% ice-cold PBS supplemented FBS three times. Endocytosis was assessed by flow cytometry and was presented as the percentage of the cells that endocytosed FITC-dextran [22]. Data analysis was performed using Navios flow cytometer (Beckman coulter life science, USA).

### Cytokine determination

The concentrations of IL-12p70 and IL-10 cytokines released by stimulated DCs were quantified by sandwich ELISA using reagents from ELISA MAX™ Deluxe Sets (BioLegend®, San Diego, California, USA) as described by manufacturer instructions. Cell culture supernatants of stimulated DCs, without or with BSEO, were collected and then filter sterilized and stored at − 20 °C. The absorbance value was estimated using a microplate reader at 450 nm within 30 min. The minimal detection level for this assay was 2 pg/mL for IL-10 and 4 pg/mL for IL-12. The absorbance value was estimated using a microplate reader (BioTek, Vermont, USA) at 450 nm within 30 min. The data were analyzed with computer-based standard curve [23].

### Microscopy

Throughout the culturing, cells were visualized and photographed using an inverted phase-contrast microscope (Nikon eclipse T¡-S, Tokyo, Japan) equipped with digital camera software (Nikon’s Digital Sight DS-U3, Tokyo, Japan).

### Gas chromatography mass spectrometry (GC/MS) analyses

BESO was analyzed using a gas chromatograph-mass spectrometer (GC/MS); Clarus 500 GC/MS (PerkinElmer, Shelton, CT] as previously described [24]. The software controller/integrator was TurboMass, 5.4.2.1617 (PerkinElmer) and Teknokroma TR-CN100 GC capillary column, (60 m × 0.25 mm ID× 0.20 μm df) was used (Teknokroma, Analitica SA, Barcelona, Spain). The column temperature program was: 80 °C hold for 5 min, increased to 150 °C (rate, 5 °C/min), and held for 5 min, increased to 270 °C (rate, 20 °C/min) and hold for 5 min. The injector temperature was 220 °C. MS scan was from 45 to 350 m/z. The total ion chromatogram was recorded from 45 to 350 m/z. The targeted peaks were extracted by the knowledge of major m/z fragments, averaged masses at the peak top, and searched for matched compounds using mass spectrometry data bank NIST2008 database. The percentage composition of the essential oil was computed by the normalization method from the GC peak area measurements [24].

### Statistical analysis

Data from at least three independent experiments from three different independent individuals were presented as mean ± standard deviation. Student’s *t*-test (Excel 2016) was used to calculate the statistically significant differences between the results. The significant difference was considered when *P-value < 0.05, **P-value < 0.01, and ***P-value < 0.001.

## Results

### Effect of BSEO on cellular viability

The cytotoxic effect of crude BSEO was determined against the HSD cell line and peripheral blood monocytes. Stimulation of cells with different concentrations of crude BSEO for 24 h showed decreased in cell viability compared to control untreated cells. The percentage of cell viability was decreased along with increased oil concentration from 5 to 50 μg/mL. HSD cells treated with 50-μg/mL crude BSEO showed a highly significant decrease in viability 52.1 ± 2.9 (*P* < 0.01). Similarly, 50 μg/mL crude BSEO induced significant decrease in monocytes viability 72.5 ± 7.3 (*P* < 0.05). Therefore, 5 *μ*g/mL and 10 *μ*g/mL concentrations of crude BSEO were selected for this study (Table 1).

**Table 1:** Viabilities of HSD cells and peripheral blood monocytes. Trypan blue exclusion assay was used to determine the viabilities of cells upon treatment with different concentrations of crude BSEO as determined by

Cells (%)	BSEO Concentrations (μg/mL)					
	Control	5	10	15	20	50
**HSD**	96.1 ± 1.6	95.1 ± 3.2	92.6 ± 5.8	88.5 ± 0.5	87.6 ± 5.7	52.1 ± 2.9**
**PBM**	96.1 ± 0.4	90.9 ± 0.2	89.3 ± 1.2	87.6 ± 4.9	84.9 ± 7.7	72.5 ± 7.3*

Data demonstrated as percentages of viability ± SD and presented as mean of three independent experiments

(*) Referred to significant value compared to control untreated viable cells, where *P < 0.05, **P < 0.01

### Effect of BSEO on monocytes differentiation into DCs

Monocytes were cultured with crude BSEO at 5 *μ*g/mL or 10 *μ*g/mL to test the ability of crude BSEO to encourage differentiation of monocytes into DCs. In parallel, monocytes were cultured either with GM-CSF plus IL-4 or 0.1% DMSO. After the incubation period, cells were investigated for DC cell surface markers. The results showed that crude BSEO did not affect cell phenotype compared to positive controls cultured with GM-CSF plus IL-4. The presence of BSEO in monocytes’ culture interfered with their differentiation into DCs. Whereas, cytokine-treated cells expressed low levels of CD14 (2.7 ± 1.5) and high levels of CD11c (97.2 ± 2.1) as typical immature DCs; in contrast, cells incubated with BSEO were significantly expressed high levels of CD14 (87.05 ± 2.6) and low levels of CD11c (5.45 ± 3.6). The expression of HLA-DR was slightly decreased in BSEO-treated cells compared to cytokine-treated cells, although no significant differences were observed. Moreover, cytokine-treated cells showed significant differences in the expression of CD86 in comparison to BSEO-treated cells and negative control cells as shown in Table 2. The expression of CD86 was also positive on the PMBCs before culturing (data not shown).

**Table 2:** Flow cytometry analysis of peripheral blood monocytes. Cell surface markers Expression upon differentiation in response to BSEO

Cell surface markers (%)	Negative control 0.1%DMSO	Differentiation inducers		
		Positive control (GM-CSF plus IL-4)	BSEO 5 μg/mL	BSEO 10 ***μ***g/mL
CD14	87.1 ± 0.8	2.7 ± 1.5	87.05 ± 2.6***	88.6 ± 1***
CD11c	8.9 ± 4.7	97.2 ± 2.1	5.45 ± 3.6***	8.7 ± 2.2***
HLA-DR	96.9 ± 0.5	99.4 ± 0.8	90.9 ± 8.9	95.5 ± 1.3
CD86	57.3 ± 2.9	8.9 ± 2.5	77.8 ± 6.9**	57.9 ± 13.4*

Data presented as mean percentages (%) of markers expression obtained from three different individuals ± SD

(*) Referred to significant value compared to the positive control, where * *P* < 0.05, **P < 0.01, *** *P* < 0.001

### Effect of BSEO on DC maturation

**Fig. 1:**
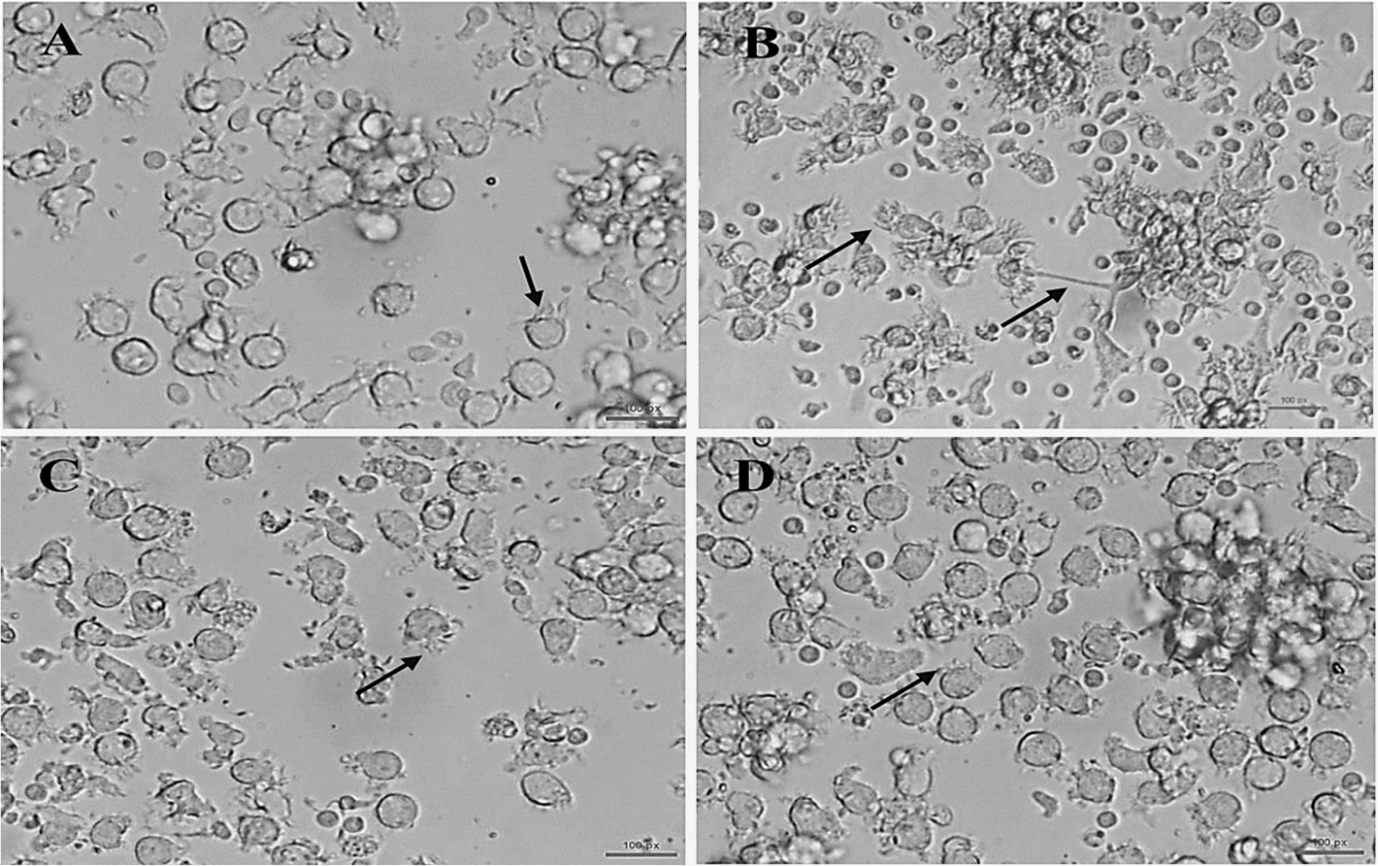
Morphology of DCs treated with crude BSEO. DCs were treated with either crude BSEO or LPS for 24 h and visualized using an inverted microscope. **a** Represents 0.1%DMSO-treated DCs that show a typical morphology of imDCs, which appeared irregular shape with short cytoplasmic projections. **b** Represents LPS-treated DCs that show a typical morphology of mDCs which seen elongated with long cytoplasmic projection. **c** Represents DCs treated with 5 *μ*g/mL of BSEO and **d** DCs treated with 10 *μ*g/mL of BSEO that exhibited imDCs features. Arrows pointed to DC projections. Photographed by phase-contrast inverted microscope (Original magnifications were 400X) at Immunology unit, KFMRC, King Abdulaziz University

To study the effects of BSEO treatment on DC maturation, cells were isolated and cultured as described previously in the materials and methods. Throughout the culturing period, cells were visualized and photographed using an inverted phase-contrast microscope. On day eight after 24 h of stimulation, typical distinctive differences of imDCs and mDCs were observed. Typical morphology of differentiated imDCs appeared with irregular shape with short cytoplasmic projections (Fig. 1a). Unlike, LPS stimulated DCs which appeared elongated and more irregular in shape with numerous and long cytoplasmic projections (Fig. 1b). However, BSEO-treated cells showed as typical as imDCs (Fig. 1c & d).

To evaluate the effects of crude BSEO treatments on the expression of cell surface markers of DC after 24 h, the percentages of CD14, CD11c, HLA-DR, CD83, CD80, CD86, and CCR7 expression were analyzed by flow cytometry. Our results showed a decreased percentage of CD14 marker on crude BSEO treated DCs and decreased expression of HLA-DR and CD11c markers similar to control unstimulated DCs and vitamin D3-stimulated DCs (Table 3). Whereas the mean percentages of cells expressing maturation marker CD83, co-stimulatory molecules CD80 / CD86, and homing receptor CCR7 were remained significantly lower at both 5 μg/mL and 10 *μ*g/mL doses of BSEO in comparison to DCs treated with LPS. These results indicated the immature status of BSEO treated DCs.

**Table 3:** Flow cytometry analysis of dendritic cells. DCs surface markers expression after stimulation with different inducers including BSEO

Cell surface markers (%)	Negative control 0.1%DMSO	Stimulation inducers			
		Positive control LPS	BSEO 5 ***μ***g/mL	BSEO 10 ***μ***g/mL	VIT- D3
CD14	2.0 ± 0.3	1.7 ± 0.4	2.7 ± 0.4	3.6 ± 0.3	92.7 ± 10.7
CD11c	94.7 ± 1.8	95.7 ± 0.2	95.0 ± 2.7	96.1 ± 0.7	94.8 ± 0.5
HLA-DR	96.0 ± 2.1	98.8 ± 0.3	89.4 ± 15.5	91.0 ± 14.2	92.7 ± 0.5
CD83	4.49 ± 0.5	82.4 ± 1.2	4.6 ± 0.9***	4.5 ± 2.3***	4.4 ± 2***
CD80	49.1 ± 7.3	97.0 ± 3.3	54.5 ± 7.5***	54.8 ± 11.9**	55.2 ± 10.4**
CD86	25.8 ± 23.7	97.1 ± 2.1	24.3 ± 15.0***	57.9 ± 13.4***	22.7 ± 21.3
CCR7	38.5 ± 19.9	73.1 ± 1.9	73.4 ± 19.4*	45.4 ± 16.4*	46 ± 16.2*

Data presented as mean percentages (%) of markers expression of dendritic cells obtained from three different individuals ± SD. Note: (*) Referred to significant difference when compared to the positive control, where *P < 0.05, **P < 0.01, and ***P < 0.001

Data in Table 4 showed that LPS stimulated DCs were expressed full maturation properties and turned into mDCs. However, stimulation with LPS in the presence of crude BSEO at 5 μg/mL or 10 *μ*g/mL did not show the same properties. The surface expression of CD86 and CD83 markers were assessed. Data indicated that the expression of CD86 was slightly reduced on DCs treated with the combined treatment in comparison to LPS-stimulated DCs. However, this reduction was not significant. Interestingly, data demonstrated that the exposure of DCs to the combined effect of LPS and BSEO (both 5 *μ*g/mL or 10 *μ*g/mL), significantly suppressed the expression of the maturation marker CD83 (*P*-value < 0.001), even in the existence of LPS compared to DCs treated with LPS alone.

**Table 4:** Flow cytometry analysis of the expression of CD83 and CD86 markers. LPS-stimulated DCs in combination with different inducers were evaluated for the expression of CD83 and CD86 markers

Cell surface markers (%)	Negative control 0.1%DMSO	Stimulation inducers		
		Positive control LPS	LPS + BSEO 5 μg/mL	LPS + BSEO 10 ***μ***g/mL
CD86	7.6 ± 2.9	96.5 ± 2.2	82.0 ± 11.8	88.7 ± 4.0
CD83	5.5 ± 1.9	82.4 ± 1.2	27.8 ± 2.2***	36.6 ± 2.2***

Data presented as mean percentages of markers expression obtained from three different individuals ± SD. Note: (*) Referred to significant difference when compared to LPS-stimulated DCs only, where ***P < 0.001

### Effect of BSEO on DC apoptosis

To determine whether crude BSEO-induce DCs apoptosis, the expression of plasma membrane phosphatidylserine was detected using the Annexin V-FITC assay. To this end, treated DCs were compared to CPT-treated DCs as a positive control for apoptotic DCs. Data in Table 5 revealed that no significant differences were found between DCs treated with any of the stimulants on the induction of early or late apoptosis compared to unstimulated controls. Whereas, CPT-treated DCs expressed significantly greater percentages of apoptosis (36%) compared to control unstimulated cells. In all treatment conditions, the viability of cells was not affected significantly.

**Table 5:** Percentages of viable, early apoptotic, late apoptotic, and necrotic DCs upon stimulation. The results shown were from three independent experiments with mean ± SD

Type (%)	Treatment					
	LPS	BSEO-5	BSEO-10	VIT-D3	CPT	Control
Viable cells	88.6 ± 1.0	88.2 ± 4.8	87.4 ± 3.9	87.5 ± 7.3	61.3 ± 5.8	88.7 ± 5.4
Early Apoptosis	*** 3.1 ± 0.8	*** 2.5 ± 1.8	*** 3.9 ± 0.9	*** 1.8 ± 1.7	29.5 ± 0.8	*** 4.7 ± 2.4
Late Apoptosis	4.7 ± 2.1	4.7 ± 1.9	5.4 ± 1.0	3 ± 1.8	6.6 ± 1.0	2.5 ± 0.5
Necrosis	2.1 ± 0.4	3.8 ± 1.2	2.3 ± 0.7	2.3 ± 0.3	3.2 ± 1.8	2.3 ± 0.9

Note: (*) Referred to significant difference when compared to the positive control treated with CPT, where ***(*P* < 0.001)

### Effect of BSEO on allogeneic T cells proliferation

**Fig. 2:**
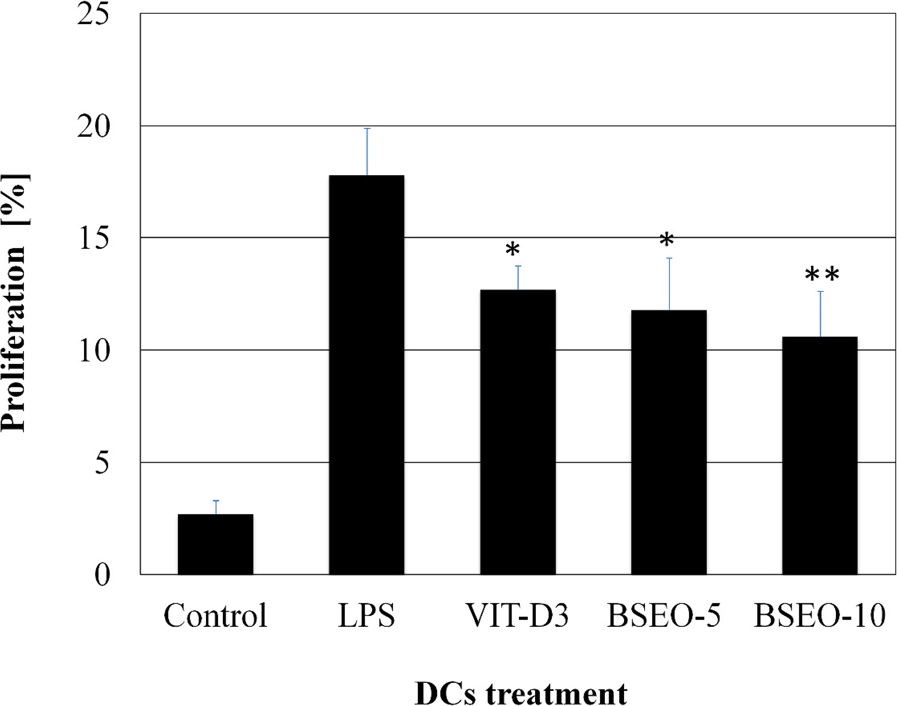
Effects of crude BSEO on T cell proliferation. Data represented the mean percentages of CD3^+^CD71^+^ T cells (± SD) co-cultured with stimulated DCs as determined using MLR assay. Results were performed from five independent experiments. Mean was significant when compared to LPS-stimulated DCs (*P < 0.05, **P < 0.01)

The ability of BSEO-treated DCs to prompt proliferation of allogeneic T cells was examined by MLR assay. The co-culture of BSEO-treated DCs with allogenic T cells was analyzed by flow cytometry. T cell proliferation capability was calculated by the percentage of CD3^+^CD71^+^proliferative T cells. Data demonstrated that the ability of BSEO-treated DCs to induce proliferation of allogeneic T cells were similar to vitamin D3-treated DCs but significantly lower (*P* < 0.01 for 5 μg/mL-treated DCs and 0.001 for 10 μg/mL-treated DCs) than the capacity of LPS-treated DCs. Interestingly, BSEO reduces the capability of T cells to proliferate even in combination with LPS when compared to cells treated with LPS alone (Fig. 2).

### Effect of BSEO on DC endocytic capacity

To explore the impact of BSEO on the endocytic capacity of monocyte-derived DCs, the FITC-dextran uptake assay was performed. LPS-stimulated DCs showed a significant reduction in FITC-dextran uptake (18.5 ± 9, *P* < 0.001) compared to control unstimulated DCs. Whereas vitamin D3-treated DCs showed no significant difference in FITC-dextran uptake compared to controls. BSEO treatments (5 μg/mL or 10 *μ*g/mL) were also unable to reduce dextran uptake by DCs, as uptake percentages remained similar to imDCs (Table 6).

**Table 6:** Percentages of FITC-dextran uptake by stimulated DCs. Data are collected from three independent experiments

Treatments	At 37 ° C (Mean ± SD)	At 4 ° C (Mean ± SD)
Mature DCs (LPS)	18.5 ± 9.0***	0.7 ± 0.2
Immature DCs (No-LPS)	64.34 ± 10.7	0.7 ± 0.5
VIT-D3	68.73 ± 7.4	0.7 ± 0.7
BSEO-5	55.1 ± 5.0	0.7 ± 0.7
BSEO-10	60.8 ± 11.0	0.4 ± 0.4

Note: (*) Referred to significant value compared to the control immature DCs (0.1% DMSO-treated DCs), where ***P < 0.001

### Effect of BSEO on IL-10 and IL-12 secretion by DCs

The immunomodulatory effect of crude BSEO on DCs was investigated by the determination of both IL-10 and IL-12p70 levels in DCs culture supernatant after 24 h treatment using ELISA assay. As illustrated in Table 7, each of LPS, vitamin D3, and BSEO (5 *μ*g/mL or 10 *μ*g/mL) stimulated the secretion of IL-10 and IL-12p70. However, BSEO-treated DCs secreted higher levels of IL-10 than LPS or vitamin D3-treated DCs. Although these increasing levels of IL-10 were not significant, however, it was significant compared to control. Alternatively, the effect of LPS on IL-12p70 secretion was significantly more pronounced than the effect of all other treatments.

**Table 7:** Levels of IL-10 and IL-12p70 cytokines. DCs cultures supernatant were assessed for cytokines production upon stimulation with different stimulant including BSEO as determined by ELISA technique. Data are collected from five independent experiments

Treatments	IL-10 pg/mL ± SD	IL-12p70 pg/mL ± SD
Control	65.3 ± 22.7	7.4 ± 1.7•••
LPS	254.3 ± 153.5 ***	318.7 ± 70.4
VIT-D3	265 ± 113.2 ***	10.4 ± 1.6•••
BSEO-5	257 ± 101.5 ***	8.4 ± 3.7•••
BSEO-10	272.3 ± 50.4 ***	10.1 ± 2.4•••

(*) Referred to significant difference compared to the control, where *** (*P* < 0.001)

(•) Referred to significant difference compared to LPS treated cells, where ••• (*P* < 0.001)

### Chemical compositions and their biological activities

**Fig. 3:**
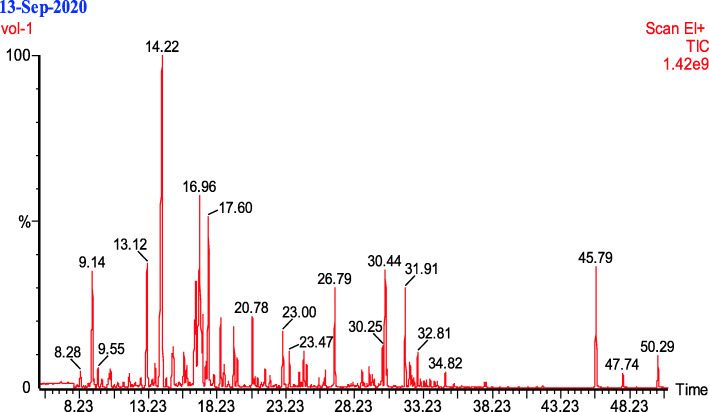
Total ion GC-MS chromatogram of characterized volatile compounds of *Boswellia sacra* resin oil extracted by hydro-distillation

GC/MS analysis demonstrates that the highest abundant compounds (Fig. 3) present in *Boswellia sacra* resin essential oil exhibit several biological activities including antibacterial, antioxidant, anti-inflammatory, antiparasitic, antineoplastic, and apoptosis and inflammatory mediators (Table 8).

**Table 8:** Compounds determined in the oil of *Boswellia sacra* resin extracted by hydro-distillation

TIME	Relative %	Compound name	Structure	Biological activity
9.14	4.91	α-Copaene		Anti-inflammatory activities [25]
13.13	5.33	(−)-β-Elemene		Anti-cancer and anti-angiogenic activities [26]
14.21	20.58	β-Caryophyllene		Analgesic, anti-inflammatory and anticancer effects [27, 28]
16.56	2.04	4-Terpinenol		Antibacterial, antioxidant, anti-inflammatory and antineoplastic activity [29]
16.68	4.55	Humulene (α-Caryophyllene)		Topical and systemic anti-inflammatory properties [30, 31]
16.96	9.51	(+)-δ-Cadinene		Anti malarial anti-microbial properties [32]
17.03	2.4	α-Selinene		Anti-inflammatory, analgesic, and antipyretic activity [33]
17.17	2.11	γ-Cadinene		Antioxidant properties [34]
17.59	6.39	β-Cubebene		Antioxidant and antimicrobial activities [35]
18.49	2.69	cis-Verbenol		Anti-ischemic and anti-inflammatory activity {Choi, 2010 #74}
26.79	3.3	γ-Muurolene		Antimicrobial and anti-inflammatory activities [36]
30.44	5.2	L-Elemol		Anti-inflammatory activities [37]
31.91	3	tau-Cadinol		Antimicrobial activity {Ho, 2011 #68}

## Discussion

Dendritic cells became promising tools involved in immunotherapeutic techniques. Down-regulation of the immune response is needed to mitigate the inflammation associated with DCs, while the upregulation of immune response requires promoting DCs differentiation and maturation. To our knowledge, no precursor data reported the impact of *Boswellia sacra* essential oil (BSEO) on human monocyte-derived DCs as professional antigen-presenting immune cells. Therefore, in this study, we assessed the outcomes of BSEO treatment on human DCs properties. The concentrations of BSEO used were based on the extent of cytotoxicity confirmed by several viability assessments. Phenotypical and functional properties have been also studied using microscopy, flow cytometry, and ELISA.

Crude BSEO is a natural product, which has chemical constituents with potential effects. Some BSEO constituents may induce toxicity to normal cells. For this reason, different concentrations of BSEO was previously tested on HSD cells and monocytes before applying on DCs. Trypan blue exclusion assay indicated that 5 μg/mL or 10 *μ*g/mL crude BSEO had no toxic effects on normal cells, HSD cells, and monocytes as DC progenitors, compared to control cells. Otherwise, high concentrations of crude BSEO at 15, 20, and 50 *μ*g/mL reduced the viability of cells in a concentration-dependent manner. Based on the above findings, less toxic concentrations 5 *μ*g/mL and 10 *μ*g/mL were used in the following experiments. Similar findings on the impact of BSEO toxicity on normal cells were also reported by [15, 24].

The ability of crude oil to induce monocyte differentiation into DCs was investigated using the two chosen concentrations. Our results showed that the addition of 5 *μ*g/mL or 10 *μ*g/mL crude BSEO to cultured monocytes did not induce complete differentiation into DCs compared to cytokine differentiated DCs. This was observed by the high expression of CD14, which is identified as a monocyte marker that usually suppressed upon differentiation into DCs. The effect of BSEO on CD14 expression, as a natural product, was also shown with a natural product chrysin, in which its suppression effect on DC differentiation from monocytes [38]. In addition, the low expression of CD11c on BSEO stimulated cells compared to control differentiated cells suggested that monocytes remained undifferentiated. In fact, CD11c is an important marker for cell attachment and always remained high on DCs [39] and usually low on monocytes.

The cell surface phenotype of DCs is used to determine the developmental and maturational status of DCs, imDCs, or mDCs [40]. The upregulation of HLA-DR, CD86, and CD83 expression on DCs is an indication of their maturation [41]. Moreover, low levels of CD83 mediate impaired T cells stimulation [42, 43]. In our hands, exposure to BSEO in the presence or absence of LPS, reduced significantly the expression of CD83, CD86, and HLA-DR on the surface of DCs, suggesting an immature phenotype of the produced DCs.

The modulation effects of BSEO on DCs maturation were comparable to the modulation of vitamin D3 on DCs, where vitamin D3 is known to block DCs maturation as previously reported [44–46]. It has also been reported the reduction of bone marrow-derived murine DC maturation treated with curcumin in the lack or presence of LPS, and which significantly reduced the expression of CD86 [47]. Both costimulatory molecules, CD80 and CD86, contribute to homeostasis and direct the immune response into immunosuppressive or immunostimulatory [48, 49]. It is well known that imDCs mediate immune tolerance, and a decrease in the expression of CD86 is a typical marker for a tolerogenic DC phenotype [50, 51]. However, CD80 has a higher affinity for CTLA-4 than CD86 and mediates immuno-regulatory responses [50, 52]. In the current study, the crude BSEO-stimulated DCs exhibited higher expression of CD80 than had CD86. We can conclude that stimulation of imDCs by crude BSEO may develop semimature or tolerogenic DCs and may mediate immunoregulatory responses. This characteristic was accompanied with lower expression of antigen-presenting molecule HLA-DR as reported by [41].

Migration of DCs to T cells areas at lymphoid tissues and organs depends on the presence of the chemokine receptor CCR7. This important role of CCR7 in migration has been verified in a knockout mouse model [53]. However, other reports have shown that, apart from chemotaxis, CCR7 controls the endocytosis level, survival, and maturation of DCs [54]. During maturation, CCR7 is upregulated to guides the migratory DCs to the nodes [55]. Independently from maturation, CCR7-expressing imDCs subsets continuously migrate to the lymph nodes to contribute to the peripheral tolerance against self-antigens even in the absence of danger signals [56].

The current study has demonstrated that DCs CCR7 expression upon addition of BSEO was significantly lower than that of LPS-stimulated DCs. Taking into account the immature status of the produced DCs, this finding may suggest that BESO-treated DCs has a teloregenic immune response. Although mature DCs use CCR7 to build up an effective adaptive immune response, imDCs use this certain receptor to migrate continuously to induce immune tolerance in lymph nodes [6]. The present study has demonstrated that CCR7 expression by BSEO-5 and BSEO-10-stimulated DCs was significantly lower than LPS-stimulated DCs. This suggest low CCR7-mediated migration and partly responsible to reduce the adaptive immune response.

The development of naïve T cells into effector cells is one of the major functions of DCs to establish the desired response, either immunity or tolerance. In this study, the determination of CD3 and CD71 expressions were used as indicators of T cell proliferation [57]. The current results indicated that addition of crude BSEO to DCs after addition of LPS has reduced the concentration of CD3^+^CD71^+^ cocultured T-cells that suggest its effect on regulating T-cells proliferation. Furthermore, the current crude BSEO showed an inhibitory effect on the ability of LPS to provoke T cell proliferation, where is similar to the effect of vitamin D3 on T cell response observed in this study. Strong evidence indicated that vitamin D3 induces tolerogenic properties of DCs, which correlate with inhibition of DC maturation, high level of IL-10 secretion, and impaired stimulatory capacity of T cells [44, 45, 58]. Therefore, this study suggested that BSEO might direct DCs towards tolerogenic ones.

Internalization is a first step required to process and present antigens in an appropriate form to prime the specific immune response [59, 60]. It is well known that imDCs are highly endocytic cells compared to mature DCs [59]. The immunomodulatory effect of BSEO stimulation on the endocytic capacity of DCs was evaluated after 24 h using FITC-dextran uptake and compared to LPS and vitamin D3 treated DCs. Data demonstrated that BSEO (5 μg/mL or 10 *μ*g/mL) increased the endocytic capacity of DCs similarly to vitamin D3-treated DCs.

Endocytic capacity of mannose receptor is strong in imDC differentiated in the presence of GM-CSF and IL-4 [22, 45]. While LPS-matured DCs express about 50% low levels of mannose receptor than present on imDCs [61]. This was also observed in our results when DCs were treated with LPS and FITC-dextran uptake was decreased.

ImDCs resemble tolerogenic DCs, which promote Treg development mediate with IL-10 production. Whereas, LPS usually induces immunogenic DCs which promote Th1 development mediated by IL-12 production [62]. IL-10 production by DCs is crucial for the generation of tolerance that suppresses the production of proinflammatory cytokines such as IL-12 [63, 64]. Furthermore, L-10-produced DCs have a role in the development of Treg immunity, whereas IL-12-produced DCs correlated to Th1 immunity [65, 66].

In the present study, cytokine analysis of DCs supernatant after crude BSEO treatment revealed a significant increase in the levels of the anti-inflammatory cytokine IL-10 secretion and reduced levels of inflammatory cytokine IL-12. Since IL-10 cytokine causes a reduction of antigen-presenting and costimulatory molecules on DCs [67, 68], this may provide a rational for the observed tolerogenic properties of DCs and supports the conclusion that BSEO regulates DCs functions and reinforces a state of immunotolerance.

In accordance to our data, it has been reported that the boswellic acids of *Boswellia serrata* inhibit the regulation of TNF-α in LPS-stimulated monocytes [69]. Moreover, the crude methanolic extract of *Boswellia serrata* and its pure component, 12-ursine-2-diketone, inhibited pro-inflammatory mediators such as TNF-α, IL-1β, and IL-6 in cultured human PBMCs [70]. Recently, several studies confirmed the therapeutic potential of some Boswellia sp. extracts and their chemical constituents as anti-inflammatory, anti-cancer, and immunomodulatory effects including antimicrobial activity towards antibiotic-resistant bacteria such as *Staphylococcus aureus* [71]. Other investigations revealed that the constituents in the oleogum resin from different Boswellia species exhibited cytotoxic efficacy against treatment-resistant human metastatic breast cancer cell line MDA-MB-231 [72]. Moreover, the boswellic extracts and 11-keto-ß-boswellic acid were also reported to suppress the activities of proinflammatory cytokines in type 1 and type 2 diabetes [73]. The analgesic activity of Boswellia and its extract has been reported to reduce osteoarthritis patients pain [74].

In the present study, we attempted to explore the major chemical components in BESO (Table 8). We found that the highest abundant constituent is β-Caryophyllen, 20% of the BESO, which is a volatile natural bicyclic sesquiterpene that contributes to the aroma of black pepper and found in many essential oils, especially clove oil, *Cannabis sativa*, rosemary, and hops. Due to its resemblance in structure and properties of cannabinoid related molecules, β-Caryophyllen binds to the cannabinoid 2 (CB2) receptor and exerts analgesic, anti-inflammatory and anticancer effects [27, 28]. However, it does not bind to centrally expressed cannabinoid receptor type-1 (CB1) or exert psychoactive effects [75]. The second abundant compound in BESO is δ-cadinene (10%), which is bicyclic sesquiterpenes that occur in a wide variety of essential oil-producing plants. Cadinene is found in several species of cotton and exerts antimalarial activity [32]. β-Cubebene constitute 6.5% of the BESO content, which is a tricyclic sesquiterpene, a constituent of the leaf oil cubebene first isolated from *Piper cubeba* berries, known as cubebs and exerts antioxidant and antimicrobial activities [35]. β-Elemen and l-Elemol are sesquiterpenoid, where β-Elemen has anticancer and antiangiogenic activities [26] and l-elemol play a role as antiinflammatory agents useful against atopic dermatitis [37]. α- α-Copaene is a complex, highly volatile, widely distributed plant sesquiterpene specially in Copaifera species that showed marked anti-inflammatory activities [25]. The next abundant compound in BESO is Humulene (4.55%), also known as α-Caryophyllene, which is a naturally occurring monocyclic sesquiterpene usually found as a mixture with β-Caryophyllen, and the two are often found together as a mixture in many aromatic plants. It has topical and systemic anti-inflammatory properties [30], and is an effective analgesic when taken topically, orally, or by aerosol [31]. tau-cadino Tau-cadinol is a cadinane sesquiterpenoid that has a role as an antimicrobial agent [76]. (S)-cis-verbenol, a natural metabolite from (−)-alpha-pinene of the host pine tree, has been found to have anti-ischemic activity [77]. Selinene is another sesquiterpene found in celery seeds that exhibits anti-inflammatory, analgesic, and antipyretic activity [33]. (−)-γ-Cadinene is a sesquiterpene and can be isolated from A. terreus. It has antioxidant properties [34]. Terpinen-4-ol, the main component of the essential oil of Melaleuca alternifolia (tea tree oil), is 1-menthene carrying a hydroxy substituent at position 4. It exhibits several biological activities including antibacterial, antioxidant, anti-inflammatory, antiparasitic, antineoplastic, and apoptosis and inflammatory mediators production [29]. The presence of these constituents with mostly antinflammatory confirms our aforementioned in-vitro results.

## Conclusion

This study focused on the immunomodulatory effects of BSEO on DCs viability, phenotype, and function. Our results showed that BSEO has anti-inflammatory properties mediated by DCs. It was demonstrated that crude BSEO acts as an immune suppressor of human peripheral blood monocyte-derived DCs, which may promote the Treg permissive environment. The suppression effects were evident in terms of differentiation, expression of maturation markers, cytokine production, and inhibition of monocyte-derived DCs stimulated with LPS. Analysis of the chemical composition of BESO revealed the presence of compounds with several biological activities including antibacterial, antioxidant, anti-inflammatory, antiparasitic, antineoplastic, properties that may explain the anti-inflammatory properties of *Boswellia sacra* resin and its oils. Altogether, this study indicates that crude BSEO may be an effective anti-inflammatory agent that requires more investigation to develop a potential therapy for many inflammatory diseases.

## Data Availability

All data generated or analyzed during this study are included in this published article.

## Abbreviations

BSEO: *Boswellia sacra* essential oil
DCs: Dendritic cells
imDCs: Immature DCs
mDCs: Mature DCs
PBMCs: Peripheral blood mononuclear cells
KFMRC: King Fahad Medical Research Center
KAU: King Abdulaziz University
